# Editorial: Fish as model organism for skeletal diseases

**DOI:** 10.3389/fendo.2023.1331690

**Published:** 2023-11-17

**Authors:** Erika Kague, Ronald Young Kwon, Christoph Winkler

**Affiliations:** ^1^ Institute of Genetics and Cancer, Centre for Genomic and Experimental Medicine, University of Edinburgh, Edinburgh, United Kingdom; ^2^ Department of Orthopaedics and Sports Medicine, University of Washington School of Medicine, Seattle, WA, United States; ^3^ Institute for Stem Cell and Regenerative Medicine, University of Washington, Seattle, WA, United States; ^4^ Department of Biological Sciences and Centre for Bioimaging Sciences, National University of Singapore, Singapore, Singapore

**Keywords:** medaka (Oryzias lapites), zebrafish (Brachydanio rerio), bone disease, osteoporosis, bone development, osteogenesis imperfecta

As our understanding of skeletal diseases continues to evolve, so does the need for innovative and versatile model organisms that can shed light on the intricate mechanisms underlying these conditions. In this context, the utilization of zebrafish and medaka, with their transparency during development, remarkable regenerative abilities, and genetic similarities with humans, provides unique opportunities to unravel the complexities of skeletal diseases. This Frontiers Research Topic on “*Fish as Model Organisms for Skeletal Diseases*” highlights the growing significance that fish species play in advancing our knowledge of skeletal development and diseases, offering new avenues for therapeutic discoveries.

## Fish provide new insights into mechanisms of rare and common bone diseases

### Rare bone diseases

Osteogenesis imperfecta (OI), also known as brittle bone disease, is a rare disease clinically characterised by short stature, skeletal deformities, low bone mass and bone fragility. In most cases, it is caused by autosomal mutations in collagen type I. Zebrafish *chihuahua (chi/-)* mutants carry a heterozygous mutation in *col1a1a* and have long been used as model for OI. Although *chihuahua* represents a very well characterised model, in which rib fractures have been described, Cotti et al. for the first-time report compression vertebral fractures associated with *chi/-.* Importantly, Cotti et al. tested whether low dietary phosphorus intake mitigates the skeletal phenotypes associated with this model, partially restoring bone shape variation of the vertebral bodies and osteoid layer of endplates. Their research suggests that reduced dietary phosphorus intake has therapeutic potential to improve bone matrix and alleviate the severe bone phenotypes of OI.

A recessive form of OI, classified as type XIV, is caused by mutations in *TMEM38B*, which encodes the trimeric intracellular cation channel Tric-b. Tonelli et al. report new insights into *TMEM38B* function. They analysed spatiotemporal expression of *tmem38a* and *tmem38b* in zebrafish, and generated a zebrafish *tmem38b* loss-of-function mutant that developed a mild bone phenotype. Interestingly, collagen type I analyses revealed enlarged endoplasmic reticulum (ER) cisternae in *tmem38b* mutants. To investigate bone formation and remodelling, they performed TRAP staining after fin amputation, and showed differential staining in mutants, pointing to abnormal osteoclast activity. Abnormal actinotrichia formation in mutants after fin amputation supported a role for *tmem38b* in osteoclast activity.

Mutations in COMP (cartilage oligomeric matrix protein) lead to chondrodysplasias. Forte-Gomez et al. revealed that *comp* is expressed in myosepta and notochord but unexpectedly not in cartilage of larval zebrafish. When they targeted *comp* using CRISPR/Cas9, irregular staining for Comp protein was detected in myosepta. Electron microscopy beautifully revealed disorganised extracellular matrix in mutants, indicating that Comp plays a role in matrix assembly, similar to what had been proposed for a subgroup of human chondrodysplasia patients. This work thus depicts the suitability of zebrafish myosepta to study extracellular matrix organisation associated with bone diseases.

Collagen II is the most abundant collagen in cartilage, however, there are many questions regarding its secretory pathway *in vivo*. Ritter et al. generated loss-of-function mutants for *rgp1*. Prior to this study, the role of Rgp1 in protein trafficking had been examined *in vitro* and in yeast, however its role *in vivo* was unknown. Using multiple approaches including *in vivo* imaging of vesicular trafficking, electron microscopy, and overexpression, Ritter et al. found evidence for a Rgp1-regulated Rab6a-Rab8a pathway that directs secretion of collagen II. This study demonstrates how the favourable genetic and imaging attributes of zebrafish enable laboratories to pursue *in vivo* studies of collagen trafficking that may otherwise be considered too challenging or scientifically risky.

### Common bone disease: osteoporosis

Other than the above-mentioned rare bone diseases, osteoporosis affects millions of people worldwide. It is characterised by impaired bone microarchitecture, commonly associated with reduced bone mineral density (BMD), and increased fracture risk.

LRP5, a co-receptor of the canonical Wnt pathway, has been associated with BMD through genome-wide association studies (GWASs). Diverse mutations in *LRP5* are causative of osteoporosis-pseudoglioma syndrome, high-bone mass, and craniofacial malformations. Khrystoforova et al. showed that zebrafish *lrp5* mutants recapitulated a loss-of-function mutation phenotype found in humans. Transcriptomic profiling of mutants surprisingly suggested the involvement of pathways implicated in osteoclast metabolism. Consistent with a role of Lrp5 in osteoclasts, they also observed increased osteoclast activity in mutant zebrafish scales. Given that osteoclasts act during fin lepidotrichia bifurcation, Khrystoforova et al. compared fins of wild-type and *lrp5* mutant fish, and found precocious fin bifurcation in *lrp5* mutants. Their work thus revealed an unexpected role of *lrp5* in osteoclasts.

Glucocorticoids (GCs) are therapeutic agents commonly used to treat immune-mediated diseases. However, their use is hampered by adverse effects in multiple organs including bone (inducing secondary osteoporosis), skin and intestine. The causes of this are not fully understood. Fleischhauer et al. surveyed the effects of GC administration in tissues with different levels of proliferation. They showed that adverse effects of GCs are particularly observed in regenerating (but not homeostatic) bone, as well as two highly proliferative tissues (skin and intestine). Because the effect of GC administration on fin regeneration mirrored effects on skin and intestine, the regenerating fin could be a useful model to understand the mechanisms underlying adverse effects of GCs in tissues with high cell turnover.

The paper by Imangali et al. provides a compelling proof of principle for a novel approach to modulate osteoclast activity *in vivo*. They used calcium phosphate (CaP) nanoparticles as carriers to efficiently deliver osteoprotegerin-b (Opgb) to osteoclasts to dampen their activity. First, the group injected fluorescently labelled CaP nanoparticles into the medaka heart, trunk or the caudal fin, showing nanoparticle stability for at least 35 hours post injection. Injecting CaP nanoparticles into transgenic reporter medaka allowed them to confidently determine CaP internalisation mostly by osteoclasts and their precursor cells, macrophages. Next, CaP nanoparticles were functionalised with a plasmid expressing Opgb specifically in macrophages, and injected into the medaka trunk muscle. These particles were successfully internalised by macrophages and expressed Opgb. To determine whether Opgb was functional, they induced bone resorption using their heat-shock inducible RankL model after nanoparticle injection. CaP nanoparticles expressing Opgb protected the bone against RankL-induced resorption.

## Zebrafish and medaka as model organisms

As zebrafish and medaka are increasingly used for bone research, understanding bone development and mineralisation in these species is essential for establishing significant comparisons with mammalian systems and confidently assessing bone phenotype in functional studies.

Miao et al. used a cathepsin K reporter to assess osteoclast location during zebrafish skull formation. Surprisingly, osteoclasts were not associated with the osteogenic fronts of active growth, but were enriched around neuromasts and their associated nerves, indicating active remodelling of the areas around peripheral cranial nerves during skull development. The authors confirmed these findings through detecting a reduced diameter of nerve canals in *csf1ra* mutants that lack functional osteoclasts.

The Notch pathway is important for craniofacial development, with Jag1 being one of its major players. Mutations in *JAG1* lead to Alagille syndrome, characterised by craniofacial malformations. Other players in the Notch pathway include *HES1*, called *her6* in zebrafish. Contrary to mice, however, *her6* mutant zebrafish do not have a craniofacial phenotype. Stenzel et al. investigated *her6-her9* double knockouts in zebrafish and discovered that *her6* and *her9* have redundant roles downstream of Jag1. Also, *her9* is vital for modulating mineralisation of the extracellular bone matrix.

In mammals and teleost models, increased bone morphogenic protein (BMP) or retinoic acid (RA) signalling causes vertebral fusions. However, the interplay between the two pathways and their targets was largely unknown. Pogoda et al. investigated the role of BMP signalling during early vertebral column formation in zebrafish. This research identified BMP as the initial pattern generator regulating the formation of the vertebral column. Using transgenic lines, drug treatment and genetic manipulations, they demonstrated that BMPs signal to notochord epithelial cells/chordoblasts to promote tissue mineralisation and notochord segmentation. Their work shed light onto the fundamental mechanisms underlying vertebral column formation in fish.

To better understand the mechanisms of bone development, it is important to standardize fish maintenance conditions and optimize imaging technologies that allow a robust assessment of the fish skeleton. Nguyen et al. used micro-computed tomography (microCT) to generate 3D models of zebrafish skeletons highlighting bone shape changes during development and in adults. With a focus on the craniofacial skeleton, this work also provides a comprehensive annotation of craniofacial bones through imaging segmentation. Standard microCTs fall short on resolution and visualisation of soft tissue. Leyhr et al. overcome these limitations by using phase-contrast synchrotron radiation microCT to image larvae and juvenile zebrafish treated with iodine-based contrast enhancer solution. They showcased the technique using wild-type and *nkx3.2-/-* mutant zebrafish, demonstrating soft tissue phenotypes in mutants. Finally, Di Biagio et al. demonstrated that fish rearing density has a phenotypic impact on the fish skeleton, independent of any genetic mutation. Medaka maintained at a rearing density greater than 5 fish/L reduced animal growth through reduction of bone mineralisation. Importantly, high density increased anomalies affecting the caudal fins and precaudal vertebral centra fusions.

## A must-read literature review: endochondral growth zones in small fish

Finally, although small fish models lack long bones, they are still able to model human skeletal disorders associated with endochondral growth zones. In a must-read review, Le Pabic et al. revisited the literature on this topic, highlighting the differences and similarities of the growth zones between zebrafish and mammals. They dive deep into explaining the growth zone organisation, contribution to endochondral growth, cartilage maturation, their replacement by bone and the importance of cartilage proliferation rather than hypertrophy to control bone size in fish. The review emphasises the use of zebrafish for investigating causes of endochondral bone diseases and the identification of therapeutics.

## Author contributions

EK: Conceptualization, Writing – original draft, Writing – review & editing. RK: Writing – review & editing. CW: Writing – review & editing.

